# Influence of Selenium Content in the Culture Medium on Protein Profile of Yeast Cells *Candida utilis* ATCC 9950

**DOI:** 10.1155/2015/659750

**Published:** 2015-06-21

**Authors:** Marek Kieliszek, Stanisław Błażejak, Anna Bzducha-Wróbel

**Affiliations:** Department of Biotechnology, Microbiology and Food Evaluation, Faculty of Food Sciences, Warsaw University of Life Sciences-SGGW, Nowoursynowska 159C, 02-776 Warsaw, Poland

## Abstract

Selenium is an essential trace element for human health and it has been recognized as a component of several selenoproteins with crucial biological functions. It has been identified as a component of active centers of many enzymes, as well as integral part of biologically active complexes. The aim of the study was to evaluate the protein content and amino acid profile of the protein of fodder yeast *Candida utilis* ATCC 9950 cultured in media control and experimental enriched selenium. Protein analysis was performed using SDS-PAGE method consisting of polyacrylamide gel electrophoresis in the presence of SDS. The highest contents of soluble protein (49,5 mg/g) were found in yeast cells after 24-hour culture conducted in control (YPD) medium. In the presence of selenium there were determined small amounts of protein content. With increasing time of yeast culture (to 72 hours) the control and experimental media were reported to reduce soluble protein content. In electropherogram proteins from control cultures was observed the presence of 10 protein fractions, but in all the experimental cultures (containing 20, 30, and 40 mg/L selenium) of 14 protein fractions. On the basis of the molecular weights of proteins, it can be concluded that they were among others: selenoprotein 15 kDa and selenoprotein 18 kDa.

## 1. Introduction

Selenium is one of those elements that determine the proper functioning of an organism by exhibiting antioxidant properties and protecting the organism against free radicals and carcinogenic factors. The primary source of selenium is a properly balanced diet, in which the food products provide the organism's need for this element. Although the content of selenium occurring in food and fodder is diverse, preparations of selenium yeasts enriched with organic forms of selenium are the most valuable and the safest methods of dietary supplements [1].

Their ingestion provides a multidirectional and beneficial effect on human health. For many years, studies on the enrichment of yeast cells with selenium have been carried out using the ability of cell biomass to bind this element. Yeast can bind ions of the elements from the environment, permanently incorporating them into their cellular structures. Development of analytical methods and the studies conducted by Schwarz and Foltz led, in 1957, to including selenium into the list of elements essential for life. This discovery initiated an extensive research on the role it plays in organisms. Despite the discovery of most of its functions, the role of selenoproteins, however, is still ambiguous [2].

Selenium is involved in the metabolism of hydrogen peroxide and lipid hydroperoxides. It constitutes an integral part of such enzymes as glutathione peroxidase (GPx), thioredoxin reductase (TR), and deiodinase iodotyrosine, which protect cells from the harmful effects of free radicals formed during oxidation processes. In these processes, selenium plays a role similar to tocopherol (vitamin E) [3]. In addition, selenium exhibits antioxidant and antitumor activity.

Selenium is a constituent of approximately 25 known selenoproteins commonly found in human organisms and 12 selenoproteins occurring in yeast cells [4]. They are involved in many metabolic processes at the cellular level. The most important biological role of selenium is associated with its presence in the active centers of many enzymes and proteins. Due to its ability to permanently bind macronutrients and trace elements into its cellular structure, yeast is not only a source of protein, but also a source of deficit microelements.

Selenoproteins were first discovered in 1973, GPx being one of them. What is more, in 1973, two additional proteins such as glycine reductase present in the cells of* Clostridium sticklandii* bacteria and formate dehydrogenase occurring in* Clostridium thermoaceticum* were discovered [5]. Selenium occurring in yeast cell is transformed to selenoamino acids, which are more absorbed from the gastrointestinal tract and additionally exhibit reduced toxicity compared to the inorganic forms.

Change of environmental conditions and occurrence of different stress factors, such as the presence of selenium in the culture medium, may lead to the expression of genes associated with the biosynthesis of new proteins in yeast cells. It is believed the expression of selenoproteins in organisms reaches a maximum value at low amounts of selenium, while, at higher doses, it may cause lipid peroxidation of cell membranes [6]. According to McKenzie et al. [7], the rate of genes transcription responsible for the biosynthesis of selenoproteins is conditioned by other abiotic factors, for example, temperature, pH, hydrogen peroxide, and hydroxyl radicals.

The metabolism of selenium compounds in cells is based on a series of transformations leading to a reduction of the degree of oxidation followed by the formation of selenide (H_2_Se). This is a common intermediate metabolite and, depending on the demand, may be used for the synthesis of selenoproteins or may be converted into methylated forms followed by their elimination from the organism in the same form [8]. Another possibility is the formation of elemental selenium in yeast cell structures [1].

The objective of the undertaken study was to evaluate the content of amino acid protein and the protein profile of fodder yeast cells of* Candida utilis* ATCC 9950 cultivated in control and experimental media enriched with selenium.

## 2. Materials and Methods

### 2.1. Yeast Strain

The strain of* Candida utilis* ATCC 9950 fodder yeast obtained from the collection of pure cultures of the Department of Biotechnology and Food Microbiology, Warsaw University of Life Science-SGGW, constituted the biological material used in the study. The strain was stored in YPD medium at pH 5.0 and at 4°C, with fresh medium being provided every 4 weeks.

### 2.2. Media and Culture Conditions

Liquid YPD medium composed of 20 g/L glucose, 20 g/L peptone, and 10 g/L yeast extract at pH 4.5–5.0 was used as the control medium. The experimental medium consisted of YPD liquid medium additionally enriched with selenium salts (Na_2_SeO_3_) in such volumes that the final selenium content was 20, 30, and 40 mg/L. Yeast inoculum was prepared by inoculating liquid YPD medium from the slant. The resulting inoculum constituted an initial material for the inoculation of the control and experimental media, 10% (v/v). Yeast cultures in the control and experimental media were cultivated at 28°C for 24, 48, and 72 hours on a reciprocating shaker with amplitude of 200 cycles/min.

### 2.3. Isolation and Determination of the Amount of Proteins

Isolation of proteins from the control and experimental yeast cell cultures was performed using Y-PER formulation purchased from Thermo Scientific (USA). Protein concentration was determined by the Lowry method [9].

### 2.4. Dialysis and Electrophoretic Separation

The resulting supernatant obtained from the isolation of proteins was subjected to dialysis (dialysis bags, MWCO 12400, 99.99%, Sigma-Aldrich, Germany) to remove all impurities (excess of salts, ions). The process of dialysis was carried out using a magnetic stirrer at 4°C for 24 hours. Polyacrylamide gel electrophoresis under denaturing conditions (SDS-PAGE) was conducted according to the Laemmli method [10] in 4% stacking gel and 20% separating gel (Mini-PROTEAN TGX Precast Gels, Bio-Rad). To determine the molecular weight, an appropriate molecular weight marker (Precision Plus Protein Dual Xtra Standards, Bio-Rad) was used. To visualize protein bands, the gels were stained with Coomassie Brilliant Blue R-250. Electrophoretically separated proteins were documented with GelDoc 2000 Gel Documentation System (Bio-Rad, France).

### 2.5. Statistical Methods

The obtained results were subjected to statistical analysis using the Statgraphics Plus 4.1. program. Significant differences between mean values were evaluated by Tukey's test at significance level *α* = 0.05.

## 3. Results and Discussion

In all cultures carried out using the experimental media, with increasing doses of selenium (>20 mg/L), the content of soluble protein slightly decreased. The highest protein content (51.7 mg/g) was found during 24 hours of cultivation in the control medium. During yeast cultivation in medium supplemented with selenium at doses of 20 and 30 mg/L, after 24 hours of cultivation, the protein content was 49.5 and 48.5 mg/g, respectively (Table 1). The obtained results did not differ significantly when comparing the content of soluble proteins in control culture biomass. Moreover, in yeast biomass obtained from the 24-hour cultivation, in the medium with the highest selenium supplementation (40 mg/L), no significant reduction in the protein content compared to the culture from the YPD control medium was observed.

In the following two days of cultivation (i.e., after 48th and 72nd hours), protein content remained at a slightly lower concentration (47.5–43.5 mg/g). Statistical analysis showed that the observed differences in the protein content obtained from a 48-hour of yeast cultivation in media supplemented with selenium at doses of 20, 30, and 40 mg/L were not significant. Similar pattern was found after 72 hours of cultivation for similar doses of selenium.

The obtained results showed that longer duration of the cultivation of experimental culture (i.e., up to 72nd hour) resulted in a reduction by about 9% of the amount of soluble protein isolated from a cellular biomass of* C. utilis* strain tested, compared to the cultivation carried out during 24 hours. For the control culture, this value was estimated at approximately 10%.

The presence of selenium in experimental media (20, 30, and 40 mg/L) and extension of the cultivation time up to 48 and 72 hours resulted in decreased protein content in yeast cell biomass in relation to the medium without any selenium supplementation. These correlations may be explained by the mechanism of the toxic effect of selenium on yeast cells. The exposure of yeast cells to selenium for a greater length of time may lead to morphological changes at the level of the nucleus (chromatin condensation followed by its fragmentation) and the whole cell. Changes were also observed in the structure of cell membranes, resulting in their increased permeability, which in consequence disrupts the functioning of channels and membrane transporters. One of the common causes of cellular protein damage is the formation of carbonyl groups caused by oxidation of amino acid residues under oxidative stress conditions. The process of protein carbonylation based on the reaction of the external amino acids with the aldehyde groups of lipids leads to the loss of cell membrane integrity [11]. The lower protein content in yeast biomass could also be the result of the detoxification processes of selenium in yeast cells: formation of the elemental form of selenium. These processes which occur in yeast cells could also be responsible for the induction of conformational changes in the polymers of the yeast cell wall [12].

Based on the studies conducted, it was found that after 24 hours of culture (after completion of the logarithmic growth phase), yeast cells began to die. As a result of the above-mentioned processes, decrease in the content of soluble protein after 48 and 72 hours of culture was observed. Probably, this phenomenon was observed as a result of the higher activity of lytic enzymes while decomposing cellular structures after completion of the logarithmic growth phase. Studies conducted by Parrondo et al. [13] showed that the longer the time of culture, the greater the decrease in the content of soluble protein in the cellular structures of* Kluyveromyces marxianus* yeasts. It was associated with progressive processes of autolysis in yeast cells in the stationary growth phase and dying yeast cells.

A progressive detoxification process occurring in the yeast could constitute another mechanism, which resulted in a reduction of soluble protein content. This process was based on the removal of protein-selenium complexes from yeast cell structures, using membrane vesicles, carrying small selenium complexes across the cytoplasmic membrane [13].

**Figure 1:**
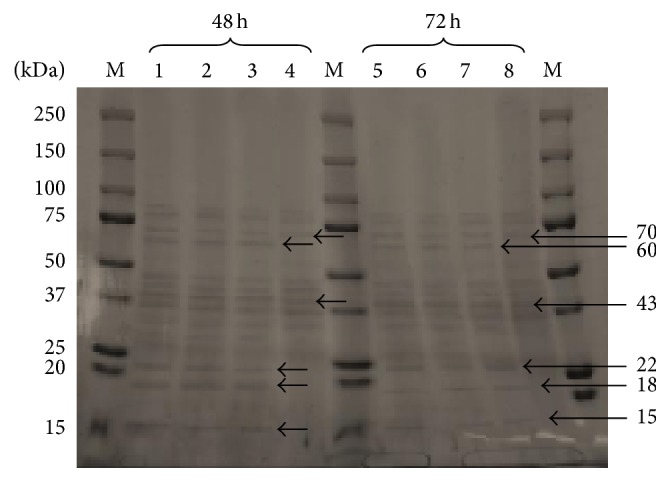
Electropherogram of proteins derived from* C. utilis* yeast biomass cultivated for 48 and 72 hours in the control and experimental media enriched in the form of Na_2_SeO_3_ (M: marker, 1: 40 mg/L, 2: 30 mg/L, 3: 20 mg/L, 4: YPD, 5: 40 mg/L, 6: 30 mg/L, 7: 20 mg/L, and 8: YPD).

The next goal of this study was aimed at a qualitative evaluation of the protein bands isolated from the yeast cell biomass of* C. utilis* in polyacrylamide gel (Figure 1). After the analysis of electropherograms, a relevant contribution of protein fraction characterized by a molecular weight of about 43 kDa in all protein extracts under study, isolated from* C. utilis* ATCC 9950 strain, was reported. According to McDermott et al. [14] and Cássio et al. [15], protein of a molecular weight of 43 kDa (Jen1p monocarboxylic transporter) is responsible for the absorption of acids, among others, lactic, acetic, and pyruvic acids. Simultaneously, this protein may be involved in the intracellular transport of selenium [14].

In the electropherogram of proteins derived from the control culture, the presence of nine protein fractions was reported, while, for proteins from the experimental culture (with the addition of 20, 30, and 40 mg/L of selenium), the number of protein fractions was equal to 14. Electrophoretic separation of yeast proteins from the control and experimental cultures did not reveal any presence of a fraction characterized by a molecular weight greater than 100 kDa. The results obtained indicate that the number of protein fractions on electropherograms was dependent on the availability of selenium in the culture medium. The resulting differences could probably be observed as a result of biochemical and physiological changes that occurred during the process of growth and aging of yeast cells.

Literature data [11, 16, 17] indicates that the presence of selenium in the environment regulates the expression of most genes involved in the response of yeast cells to oxidative stress (mainly via Yap1p transcription factor, glutathione reductase, and glutaredoxin), which in consequence could affect the expression of genes involved in protein synthesis.

The occurrence of four new fractions on the electropherogram of proteins derived from yeast cells cultivated in media supplemented with selenium indicated a significant influence of this element on the biosynthesis of selenium proteins in yeast cells. It is worth noting that the presence of new protein fractions did not affect the increase in the total content of soluble protein in yeast cells cultivated in media supplemented with selenium. Such correlation may result from the small content and the molecular weight of protein fractions obtained as well as from progressive detoxification processes in yeast cells.

Studies presented by Błażejak [18] demonstrated the presence of seven protein fractions isolated from yeast cell biomass of* C. utilis* cultured in control YPD medium. Differences in the number of protein fractions obtained in the above-mentioned study in the control YPD medium (14 fractions), when compared to the data presented by Błażejak [18], resulted from the additional dialysis of protein extracts isolated from yeast biomass. The use of commercial polyacrylamide gels (20%) was also reflected in the higher amounts of protein fractions that were obtained. The general pictures of the proteins extracted from yeast cell biomass originating from all cultures supplemented with selenium, in principle, did not differ from each other. The only observable differences in the number of the fractions obtained could be observed between the proteins isolated from yeast cell cultured in the control (YPD) and in the selenium-supplemented media.

Analysis of electrophoretic separation of cellular proteins obtained after 24 hours of experimental culture revealed the presence of new fractions of molecular weight 22 and 60 kDa, which were not found in proteins from the control culture. According to the literature data [19], the first protein present in yeast cells of similar weight is the complex of GPx enzyme (glutathione peroxidase). GPx, one of the first selenoproteins to be characterized, usually exhibits a tetrameric structure with one selenium atom in each subunit. So far, eight types of GPx have been identified, of which five occur in selenocysteine. In terms of a protein characterized by a mass of 60 kDa, it could be selenoprotein P, which contains up to 10 selenocysteine residues [20]. Increased activity of this enzyme complex in the cells of the* C. utilis* strain under study could result from the presence of selenium ions, which in the experimental medium are responsible for the occurrence of stress conditions, among others, oxidative stress [10, 21].

In addition, the presence of the protein fraction of 70 kDa was observed in yeast cells from both the control and the experimental media, regardless of the duration of culture time and selenium concentration. According to the literature data [22], it could be TR (thioredoxin reductase) that contains a selenocysteine sequence in its catalytic center. Proteins isolated from* C. utilis* yeast cells cultivated in media supplemented with selenium (20, 30, and 40 mg/L) contained more protein fractions of molecular weights below 30 kDa when compared to cells from the control YPD medium. It proved the occurrence of transport processes and bioaccumulation of selenium yeast cell structures. Based on the obtained results on molecular weights of proteins, it can be concluded that they involved 15, 18, and 22 kDa selenoproteins, respectively [22].

According to Tastet et al. [23] and Schaumlöffel [24], the protein characterized by molecular weight of 18 kDa is SIP18 (salt induced protein 18). The literature data [25] indicates that biosynthesis of this protein is activated under environmental stress conditions in the experimental culture in the presence of salt (in this case, an excess of selenium IV ions).

Extension of the duration of yeast culture up to 72 hours in all media (control and supplemented with selenium) did not affect the number of protein fractions isolated from* C. utilis* ATCC 9950 yeast biomass. In the studies conducted by Chassaigne et al. [26] on commercial lyophilisates of* Saccharomyces cerevisiae* selenium yeast containing 1300 *μ*g/g of selenium (Pharma Nord, Vejle, Denmark), the presence of 15 intensive protein fractions was demonstrated. The highest intensity was observed for molecular bands of 30 kDa. Using ETV-ICP-MS (ICP-MS with electrothermal evaporation), the authors were able to determine the concentration of selenium in the individual protein bands.

The highest content of selenium was found in the protein fractions characterized by molecular weights of 14 kDa (10 ng/cm gel), 25 kDa (approximately 5 ng/cm gel), and 43 kDa (12 ng/cm gel). Above the molecular weight of 100 kDa, no protein fractions were reported. Different results were presented by Bryszewska et al. [27], who demonstrated that high accumulation of selenium in* S. cerevisiae* yeast cells occurred in protein fractions above 75 kDa.

Interesting observations in this regard have been made based on the results of the studies conducted by Zhang et al. [28]. In proteins of* Bifidobacterium animalis* 01 from the control MRS medium, as well as from medium supplemented with selenium at a dose of 2.5 mg/L, the presence of 16 protein fractions was demonstrated. It was found that selenium was present in all protein fractions isolated. The highest content of this element was observed in bands that corresponded to a molecular weight between 10 and 20 kDa (24 ng/cm gel). The analysis of the results showed that more selenium was involved in interactions with proteins of low molecular weight.

The results of electrophoretic separation of proteins isolated from* C. utilis* yeast cells cultivated in media supplemented with selenium, in relation to those obtained from the control media, exhibited the presence of four new protein fractions characterized by weights of 60, 22, 18, and 15 kDa. It proved the activation of processes associated with metabolism of this element. Several authors [29, 30] suggest that the presence of selenium in the culture medium effected the changes in the antioxidant system of yeast cells, which resulted in an increased protein biosynthesis of selenium-dependent proteins.

In summary, the observed differences in the protein profile obtained from yeast biomass derived from both the control and the experimental cultures supplemented with selenium (20, 30, and 40 mg/L) indicate the possibility of expression of genes responsible for the synthesis of selenoproteins, among others, GPx (22 kDa). The contribution of selenium to the metabolism of* C. utilis* ATCC 9950 yeasts is associated with protection against oxidative stress in cells. The presence of selenium in the culture medium induces the activity of many enzymes associated with intracellular transport of selenium; however, these processes did not affect the increase in the content of soluble protein in yeast cells. The analysis of electrophoretic separations demonstrated qualitative differences between the protein bands obtained from yeast cells cultivated in the control and the experimental media. In protein fractions of yeasts obtained from media supplemented with selenium (20–40 mg/L), the presence of new protein fractions characterized by molecular weights of 60, 22, 18, and 15 kDa was revealed. The presence of selenium in the medium affected the occurrence of oxidative stress, which in turn induced the expression of genes responsible for the biosynthesis of selenoproteins in yeast cells.

## Figures and Tables

**Table 1:** Changes of protein content (mg/g) isolated from yeast cells *C*. *utilis* grown in control (YPD) and experimental media enriched with selenium.

Selenium contents in medium (mg/L)	Cultivation time (h)		
	24	48	72
	Protein content (mg/g)		
0 (YPD)	51,77 ± 2,58^d^	47,53 ± 1,60^abcd^	46,77 ± 1,57^abc^
20	49,52 ± 2,00^cd^	46,95 ± 2,00^abc^	46,02 ± 0,93^abc^
30	48,57 ± 1,91^bcd^	44,44 ± 1,07^ab^	43,79 ± 1,22^a^
40	48,08 ± 0,64^abcd^	43,73 ± 0,74^a^	43,51 ± 1,76^a^

Values marked by the same letter are not different at *α* = 0,05.
